# Qualitative assessment of patients’ attitudes and expectations toward BCIs and implications for future technology development

**DOI:** 10.3389/fnsys.2015.00064

**Published:** 2015-04-27

**Authors:** Silke Schicktanz, Till Amelung, Jochem W. Rieger

**Affiliations:** ^1^Department of Medical Ethics and History of Medicine, University Medical Center GöttingenGöttingen, Germany; ^2^Department of Psychology, University ofOldenburg, Germany; ^3^Research Center Neurosensory Science, University of OldenburgOldenburg, Germany

**Keywords:** brain–computer interface (BCI), patient attitude, ethics, interviews, empowerment, medical risk, privacy, acceptance

## Abstract

Brain–computer–interfaces (BCIs) are important for the next generation of neuro-prosthesis innovations. Only few pilot projects have tested patients’ abilities to control BCIs as well as their satisfaction with the offered technologies. On the one hand, little is known about patients’ moral attitudes toward the benefit-risk-ratio of BCIs as well as their needs, priorities, and expectations. On the other hand, ethics experts intensively discuss the general risks of BCIs as well as the limits of neuro-enhancement. To our knowledge, we present here the first qualitative interview study with ten chronic patients matching the potential user categories for motor and communication BCIs to assess their practical and moral attitudes toward this technology. The interviews reveal practical and moral attitudes toward motor BCIs that can impact future technology development. We discuss our empirical findings on patients’ perspectives and compare them to neuroscientists’ and ethicists’ perspectives. Our analysis indicates only partial overlap between the potential users’ and the experts’ assessments of BCI-technology. It points out the importance of considering the needs and desires of the targeted patient group. Based on our findings, we suggest a multi-fold approach to the development of clinical BCIs, rooted in the participatory technology-development. We conclude that clinical BCI development needs to be explored in a disease-related and culturally sensitive way.

## Introduction

Brain–computer interface (BCI) research is a highly interdisciplinary field that emerged between the 1930s and 1970s (Hess, 1932; Delgado, 1969; Vidal, 1973). The goals of BCI development are manifold, but one focus is clearly on the development of devices that support or replace neural function for easing daily life of people with neural dysfunctions (Lebedev and Nicolelis, 2006; Lebedev et al., 2011; Quandt et al., 2012a,b; Wolpaw and Wolpaw, 2012). BCIs can be divided into two classes with respect to the direction of data flow between the neural and the technical system. The first type, the readout-BCI, measures and analyses brain activity in order to infer intentions, deliberating acts, perceptions, changes in cognitive states etc. from short intervals of data (Rieger et al., 2008; Hollmann et al., 2011; Hochberg and Anderson, 2012; Guger et al., 2013; Reichert et al., 2014). This is the type most authors in neurosciences currently refer to when using the term BCI. Users can control these devices by actively producing changes in neural signals. Readout-BCIs could in principle assist users to communicate, control prostheses or wheelchairs, support rehabilitation, or facilitate detection of consciousness. The aim is to make these technologies potentially useful to those with paralysis (Nuffield Council on Bioethics, 2013). The second type, the stimulation-BCI, alters brain activity in order to modify intentions, deliberate acts, perceptions, cognitive or emotional states etc. Until recently, both lines of research were fairly independent of each other.

For readout-BCIs the most important fields of development aim at (prosthesis) control and communication. The targeted population consists of patients with severe motor deficits caused by degenerative diseases of the motor system (e.g., (Amyotrophic lateral sclerosis, ALS) or injuries (e.g., spinal tetraplegia). The intended BCI seeks to restore or ease communication with family and caregivers (e.g., ALS), to provide means for autonomous mobility, e.g., wheelchairs (e.g., paraplegia; Carlson and del Millan, 2013) or exoskeletons (Fitzsimmons et al., 2009), and to re-establish some autonomy, for example, by controlling a robotic arm for grasping and self-feeding (e.g., for tetraplegia; Velliste et al., 2008). Research in these core fields has been driven almost exclusively by engineering questions (e.g., How can we involve more degrees of freedom into control? How can we increase the bit-rate?). There have been several impressive demonstrations of applications, such as a letter from an ALS-patient to the principal investigator written with a non-invasive BCI-speller (Birbaumer et al., 1999), or a woman feeding herself via an invasive BCI that controls a robotic arm (Hochberg et al., 2012). However, despite intense research efforts, to date, only a surprisingly small number of patients seem to be using readout-BCIs on a regular basis. Hochberg and Anderson (2012) estimate the worldwide number of patients using readout-BCIs on a daily basis to be less than ten. Therefore, the readout-BCI must still be considered a research approach rather than a therapeutic treatment (Nuffield Council on Bioethics, 2013). In our own empirical ethical study we focus on readout-BCI, but we think that for the following initial assessment of the status of BCI technology it is helpful to include stimulation-BCI.

Compared to readout-BCIs, several stimulation-BCIs have been extremely successful and are now widely used in the clinical context. The cochlear implant (CI) can be considered a hallmark which the NIH estimates to be implanted in approximately 324,000 patients worldwide by 2012 (cited from Clark, 2014). Similar to most readout-BCIs the CI system requires adaptation of the user to the technical system in order to understand speech. Furthermore, it requires socio-cultural acceptance by the deaf community as well as an individual benefit assessment (Blume, 2009). Deep Brain Stimulation (DBS) is another stimulation application, which is now widely used to reduce key symptoms of motor diseases and even cognitive dysfunctions (Kringelbach et al., 2007 for a review). Recently, BCI research on motor prosthesis has begun to focus on providing somatosensory feedback through stimulation-BCI to improve dexterity of prosthesis control (O’Doherty et al., 2011; Dadarlat et al., 2015). Like CIs, somatosensory stimulation will require invasive approaches to achieve a sufficient level of detail of the evoked sensations.

This raises the question why some BCI approaches are more successful than others. To date, stimulation-BCIs have found much wider application than readout-BCIs. Apparent reasons include the difference in invasiveness and the potential cases of application. Although much research with animals showed the superiority of invasive readout-BCIs with regard to performance, most human readout-BCI researchers do not have the infrastructure required for such developments or consider invasive devices unacceptable in humans. Other apparent differences are, that so far readout-BCIs in general were mostly developed using a healthy young population, which is quite different from the actually targeted patient population, the experiments had a limited duration, and the subject’s motivation was limited to a short time interval. In such settings, researchers lack valuable feedback on the usefulness of the implemented approach from potential users. Thus, they bear the risk of producing results that are not applicable to or accepted by the target population.

In practice, only few groups went through the time-consuming process of extensively training patients of the target population on readout-BCI control; some of those who did were able to train their patients to achieve an impressive performance (Birbaumer et al., 1999; Hochberg et al., 2012). Even the most successful stimulation-BCI, the CI, requires months of training until patients are able to understand speech. Development of speech comprehension is delayed even for children who received implants pre-lingually before the age of two (Svirsky et al., 2004). Another significant difference between readout- and stimulation-BCIs is that the latter were developed as a last resort for patients who severely suffered from their deficits without having an acceptable alternative treatment or technologies at hand. Most readout-BCI paradigms require motivation to achieve control which is likely high in such patients but they can also produce frustration if the device is not useful.

Despite the huge increase of readout-BCI research, paraplegic or ALS-patients, who depend on daily care, benefit to date less from BCI development than deaf patients do from CI, who can still live an independent life. Thus, it appears extremely important to leave it not only to the developer to decide what handicap is so severe that BCI is an ultimo ratio or what could be a useful paradigm. When developing readout-BCIs it seems very important to take both into account: the technical aspects and the needs and desires of the targeted group of people.

What is missing is an ethical–practical perspective collecting, reflecting, and assessing the complex experiences of affected persons such as patients with tetra- or paraplegia with regard to BCIs. Such studies are very rare and often focus on the functional assessment of pilot studies but neglect the broader context of such patients. For example, Huggins et al. (2011) conducted a quantitative multiple-choice interview study via telephone with 61 ALS-patients (for spinal cord patients, see e.g., Snoek et al., 2004). A few pilot studies have evaluated BCI prototypes for communication, rehabilitation and robot arms in the US, Romania, and Germany with regard to patients’ satisfaction or disappointment, ability to control the BCI, potential side-effects, and data protection (Kübler et al., 2005; Nijboer et al., 2008; Onose et al., 2012; Vaughan et al., 2012; Grübler et al., 2014). Such studies can be seen as an important, even mandatory requirement (Hochberg and Anderson, 2012) as able-bodied researchers may not be able to fully anticipate the concrete needs and wishes of a medical BCI from a patient’s perspective. To our understanding, evaluating and analyzing an affected person’s perspective goes beyond a simplistic individual case-based, risk-benefit analysis. This is because it takes account of the socially embedded experiences of such patients within a particular techno-social culture. The term ‘techno-social’ indicates the close interrelatedness and co-existence of a technology with social rules, norms, and practice (Beck, 2000). The cultural background (here Germany) can strongly influence a target group’s experience considering stigma, social health care provision, and public acceptance of human–machine interfaces. Furthermore, there is a particular professional responsibility for how such a medical device is developed, as professionals and researchers should take on the role of a gate keeper, or responsible door-opener for new technologies with substantial social implications.

## Methodology

To gain a better understanding of individual and social expectations toward BCIs, we collected data from affected persons with different but significant types of mobility-disabilities. By applying open interview methodology, we aimed at collecting evaluations and assessments of the practical, social, and moral issues of readout-BCIs. Such a perspective is embedded in the complex interaction of one’s own body, the social environment and the personal biography of the affected person, an issue difficult to reveal with quantitative surveys. We wanted to explore how the patients’ understanding of their disability and their experience with existing human and technical support are important for the contextualisation of the interviewees’ positions. This qualitative interview study design allows data driven formation of hypotheses. The study received approval by the Research Ethics Committee of the University Medical Center Goettingen (no. 12/6/13).

### Recruitment and Sample

Potential interviewees were recruited between April and July 2013 via flyers sent out to various self-help groups and clinics for persons with bodily disabilities and muscle disease. These networks cover a broad regional spectrum from Central-West to North–West Germany. Self-recruitment (as opposed to recruitment through doctors and clinics) was encouraged to avoid biased positions, to include patients not yet enrolled in clinical BCI trials. We hoped to obtain more general attitudes toward this innovation from such patients.

We included four patient types as interviewees who are considered potential future users of motor and communication readout-BCIs, namely patients with ALS, muscle atrophy, para- and tetraplegia. We introduced readout-BCIs for restoring or controlling motor function to them by providing a short video sequence (see below). We chose motor readout-BCI because it is a major field of research.

We conducted open question interviews with ten people for this study. All interviews were conducted in German language at the interviewee’s private home and lasted between 0.5 and 2.5 h. The gender-balanced sample covers a spectrum of different disabilities (see **Table 1**). One out of the 10 interviewees was a female caregiver who intensively cared for her recently deceased father who had ALS. She herself was not considered as ALS patient, but argued very empathically and in a way as she was at risk for the disease and was therefore included into our sample. All interviewees lived in their own house or flat with long-term assistance from one or more family members or a professional caregiver (which is covered by health care provisions in Germany).

**Table 1:** Anonymized sample of interviewed patients.

Code	Gender	Age	Profession	Disease/disability [relevant for Brain–computer–interfaces,(BCI)]	Assisted by
F1BM	Female	40	Social worker	Spinal muscular atrophy Type 2	Several personal assistants
F2BM	Fetale	38	Unemployed	Spinal muscular atrophy Type 2	Mother and professional nursing service
F3BM	Female	50	Office clerk	Spinal muscular atrophy Type Kugelberg-Welander	Several family members as caregivers
F4A	Female	50	Medical technical assistant	Caregiver (unclear whether ‘at risk’ for Amyotrophic lateral sclerosis, ALS); her father had ALS (recently deceased)	Herself, as daughter, was long-term caregiver for her father
M5BM	Male	70	Engineer (retired)	ALS	Wife
M6BQ	Male	50	Head of an assistance service	Tetraplegia	Several personal assistants
M7BM	Male	Unknown	Unknown	ALS	Mother and professional nursing service
M8BV	Male	56	Local politician (retired )	Paraplegia after accident and possible error in treatment	Several family members as caregivers
F9BQ	Female	5	Sociologist, university professor	Paraplegia	Household help
M10BP	Male	60	Psychologist	Post-Polio-Syndrome	Household help, friends, personal assistant

### Data Collection and Analysis

Interviews were conducted according to the problem-centered approach (Witzel and Reiter, 2012). This methodology follows an open question, narrative structure to encourage interviewees to set their own foci. As initial starter, we presented a short video sequence showing the chocolate nibble taken by Ms. Jan Scheuermann by using a readout-BCI controlled robot arm. This readout-BCI was implanted and conducted at the University of Pittsburgh. It was initially shown on the US “NBC News” (December 17, 2012) publicly available on “YouTube” [http://www.youtube.com/watch?v=C7H_M8-dBHc (latest access: 30 Dec 2014]. The English video was shown to the interviewees with German subtitles. After the viewing of the video, the interviewees were encouraged to comment and assess the research from their perspective. None of our interviewees had any previous personal experience with BCIs. Their medical–technical knowledge of BCIs was rather limited. Only half of the interviewed persons had heard about BCIs before. In general, the video input was considered helpful for understanding the basic idea of motor readout-BCIs, and hence interviewees commented mainly on BCIs we classify as readout-BCI. In case the interviewees did not bring up topics by themselves we additionally used the following open question guide for comments:

(1) Would you ever use a BCI and if so, under which circumstances would you do so?
(2) What are your experiences with former and your current care takers?
(3) What are your experiences with former and your current technical devices?
(4) What are your expectations of medical practice and research?
(5) How do you gain information about innovations in medical and engineering research that might be of personal relevance for you?

The interviews were audio-recorded and consequently transcribed. All material was pseudo- anonymised for further analysis and anonymised for publication. The analysis was firstly done in German language to ensure hermeneutic accuracy with regard to metaphors and phrases. Results were later translated into English for publication.

The material was analyzed by means of content analysis (Mayring, 2010) with the scientific software Atlas.ti^TM^. The content analysis of the coded material included summarizing major positions and clustering findings according to more general lines of argumentation (see ‘Results’ below). Coding and categorizing of statements was carried out in order to identify general lines of argumentation and topics. The following five major codes were seen as most effective for categorizing the interview statements: (A) general attitudes toward BCIs; (B) attitudes toward and experience with personal assistance; (C) attitudes toward and experience with technical assistance; (D) expectations toward modern medicine; and (E) self-and external perception of disability. The results section below is organized along the codes. In order to increase coding accuracy, the coding process was confirmed by independent peers not directly involved in the project.

## Patients’ Interview Findings

In the following, we will present the major findings with regard to the lines of argumentation and positions of the interviewed patients. We focus in our analysis mainly on practical attitudes (everyday-life conformity and how to handle things) as well as on moral attitudes. By moral attitudes we mean all kind of statements related to moral relevant issues of what should be allowed, forbidden, protected, or promoted. Statements mentioning moral principles were rarely explicitly made (e.g., use of terms such as ‘autonomy,’ ‘justice,’ ‘beneficence,’ ‘non-harming,’ ‘respecting vulnerability’ etc.) but we identified various moral values on an implicit level via content analysis. Therefore, it is the aim of our empirical-ethical analysis to detect and condense such moral attitudes for further discussion and reflection.

Despite the low pre-existing knowledge of BCIs within our interview group, we found very comprehensible and detailed considerations, as for example, no interviewee reacted irrationally or showed strong emotions such as disgust about the concept of BCI. We observed no influence of the extent prior-knowledge on the interviewee’s position.

The arguments tagged by code “general attitude to BCI” are clustered according to their positive (proponents’) or critical (skeptics’) assessment. Furthermore, we identified ambivalent statements which present concrete (and a few general) concerns. The arguments in these three clusters will be presented in the following three sections. From the fourth section “Factors Influencing Attitudes” on we analyzed the factors influencing the attitudes determined by the other four codes.

### Proponents: “Increasing Personal Privacy and Reducing Dependence”

The six patients in favor of BCI research had spinal muscular atrophy, ALS, or tetraplegia. They particularly expressed their hope of gaining more self-control and independence in basic, everyday activities. This is considered the ‘right to privacy’ in philosophy and law. It includes the right to be alone, without any unwanted intrusion and the right to experience solitude. In particular, intimacy and activities related to personal hygiene were frequently mentioned as part of personal privacy. As a consequence, BCIs are expected to reduce the dependence on and need for human assistance, which was a requirement in daily routine for all interviewees. Moreover, both the interviewed ALS-patients as well as the related caregiver of the ALS-patient stressed the need for a communication BCI. Improving or ensuring communication was seen as a fundamental issue, because the serious impairment of communication abilities was interpreted as a reason for personal suffering. Restoring or enabling communication for those affected by ALS was regarded as important for increasing quality of life, for avoiding social isolation or even for maintaining the will to live. Apart from the generally positive attitudes, some interviewees critically assessed the medical risks linked to the necessary surgery for invasive BCIs. Especially anesthesia is regarded as a high medical risk factor in surgeries performed in patients with ALS or muscle atrophy related to neuro-inflammatory processes and should be avoided. Therefore, non-invasive solutions or low doses of anesthesia are preferred.

### Skeptics: “Insufficient aims, Stigmatization, and Data Insecurity”

Patients whose disability did not require permanent personal assistance tended to make negative assessments. Critique focused on practical technical aspects. Concerns for invasive BCIs included the adequate relation of personal benefits on the one hand and medical risks and limits to data protection on the other hand. One interviewee with paraplegia criticized the hype concerning the success of Jan Scheuermann and questioned whether eating independently actually is of interest for such a person. Instead, moving and lifting one’s own body independently was regarded as more important in the skeptical group. One patient expressed concern about data security (low technical protection or leaking of personal data) and the risks of misuse (e.g., illegitimate access and instrumentalisation of personal data on behalf of third party interests). The interviewee asserted that BCIs could be used to monitor patients’ activities or even thoughts. These concerns were embedded within the rather general opinion that data insecurity and misuse should be seen as an increasing public phenomenon: too many personal data are collected by the state, and the use of surveillance technology in public impairs citizens’ rights to information privacy. Another patient expressed concern that BCI technology could lead to neglecting existing mobility and demotivating people to do regularly exercise to keep one’s muscles active. This concern was clearly related to the video input as BCIs for rehabilitation purposes were previously neither discussed nor known by this patient. Finally, a purely ‘medical view’ on disability was criticized. Such a view, as the skeptic said, neglects the social factors regarding dependence and stigmatization. This is the case when BCI approaches are contextualized as a cost reduction of care work or as to increase mobility by ‘walking’ because ramps are still missing in many buildings.

### Ambivalent Attitudes: “Trade-off Between the Caregiver and Technological Dependence”

Those who argued in support of BCI technology were not without criticism. Most of them expressed the fear that BCIs are hyped in the media but are still not in sight as a real future option. In addition, patients feared that complex BCIs would not lead to more self-determination, independence and privacy but could create new dependencies (e.g., from technical service). Furthermore, the wish for a realistic depiction of BCIs for patients was stressed, as it was regarded as unrealistic to compare seriously disabled persons with able-bodied persons through the application of BCIs. In reference to the case of Jan Scheuermann, one interviewed person asked ironically how the chocolate came so close that she could take a bite. Only one patient problematized that BCIs might transcend our self-understanding as human beings by crossing humans with machines. He referred to the metaphor of “Frankenstein’s monster” to express his personal ambivalence but did not weigh such a ‘feeling’ as sufficiently important to reject technological innovations.

### Factors Influencing Attitudes

The patients’ experience with human as well as technical assistance was important for understanding their respective positions. Experience with human assistance was vital for understanding positive attitudes and concrete hopes attached to the functions of potential BCIs for daily life. Experience with technical assistance was essential for understanding concrete concerns about functionality and doubts about clinical practicability.

#### The Human Factor: Interpersonal Challenges

In Germany, various models of human assistance exist and are financially supported by public health services. This includes care services at the home or in assisted living, one or more relatives as caregivers, and the so-called personal assistance. Personal assistance is particularly important, as this labor relation model allows the affected person to select and train a person as his/her personal assistant (Kotsch, 2012). This model is generally seen as the most empowering. However, as the analysis of the code “general attitudes toward human assistance” revealed, most of the negative experiences expressed by the patients were related to this model. This included dissatisfaction with the execution of commands as well as the not always harmonious cooperation between assistant and employer. Moreover, the dependence on another person was sometimes experienced as a social burden as the employer–employee relationship continuously required emotional control and the need for social commitments (such as politeness). It also often compromised the patient’s privacy. In concrete care situations, during stays in nursing homes or hospitals, the affected persons experienced situations where time is limited, routine prevailed, and choices were lacking. The personal bodily contact with human assistants was not always described as pleasant by the patients and sometimes even as intrusive. In the context of such experiences, the development of technical assistance or replacements of humans with robots was regarded as very advantageous.

#### The Technical Factor: User-Centered Design for Daily Activities

All interviewees had intensive experience with the use of technical assistance, which is essential for accomplishing their daily routine. The spectrum of assisting measures was rather broad, including visual and hearing aids, prosthesis, electric wheel chairs, or electric bed lifts. The results in this section are based on the analysis of the codes “general attitudes toward technical assistance” and “toward modern medicine.”

Technical assistance was often embraced and seen as true empowerment. One patient criticized the phrase of “to be wheel chair bound.” For her, the electric wheel chair was a rescue and liberation. Technical progress is therefore appreciated, especially in everyday practices such as listening to music, opening doors and windows, controlling the heating, etc. Importantly, disappointment and dissatisfaction with technical assistance is expressed in cases where technical assistance is developed by means of a deficit model of disability. For example, the older models of wheel chairs were built for the use in hospitals and inside of homes but are not sufficiently robust for outdoor activities, as one interviewed patient described clearly. The interviewees interpreted this issue as a social practice of exclusion, judging disabled persons as being disinterested in or even not worth going outside – into the public.

Overall, technical assistance was appreciated if it was valuable for the daily routine. Great satisfaction with existing technical assistance tended to support disinterest in BCIs and vice versa. Another important point was that fair access to advanced technical assistance was regarded as critical because of current political tendencies of rationing in health care. Some also expressed their experience that existing technical assistance is unfairly distributed and often only made available to people with a high socio-economic status.

### Self- and External Perspective on Disability

Under the code “self- and external perspective on disability” some interviewees expressed strong concern about the social identity given to persons with disability. According to their perception a particular image of disability prevails within medical sciences, healthcare, and the public. Disability is interpreted as a ‘deficit model’ meaning that the patient’s life is depicted in a rather negative way by setting the focus on activities the patient is unable to do but are considered as ‘normal’ and ‘necessary’ (e.g., walking, eating by using hands). Some reported how they experienced the expression of pity or taboos by others about their disability as stigmatizing or discriminating. This social, external perspective was contrasted with the own perception of the interviewees. Most of them expressed ways of resilience, showed high self-esteem and reported about their satisfaction when managing successfully challenges of private or work life.

In sum, 6 out of 10 interviewees very positively assessed readout-BCIs, while two were very skeptical and two were ambivalent (see **Table 2** for a brief overview of interviewees’ major lines of arguments). We found a negative interrelation between the degree of approval and the degree of satisfaction with human, personal assistance. The more patients were dissatisfied with human assistance, the stronger were their positive expectations toward BCIs. Especially, interviewees with very serious disabilities regarded BCI technologies as a form of empowerment. Individual experience with human and technical assistance was crucial for the assessment of potential benefits and risks of readout BCIs. All interviewees had experience with existing-types of technical assistance and construed hopes and fears about feasibility and risks from BCIs. Readout-BCIs were regarded as attractive by patients if they were to provide them with more privacy and intimacy. The more experience a patient had with advanced technical assistance, the more detailed concerns about error-rates and realistic risks of a malfunction were expressed. Finally, all patients stressed the need to ensure fair access to such health care technologies. Most had already experienced difficulties in receiving sufficient financial support for technical and personal assistance to support them in their active everyday-life.

**Table 2:** Overview of general attitudes revealed during the interview by the interviewees.

Code	Gender	Disease/disability	Summary of attitudes toward BCI
F1BM	Female	Spinal muscular atrophy Type 2	Very positive; very strong desire to reduce human assistance; general positive attitudes toward technical devices; but also concrete concerns related to her medical condition connected to invasive BCI
F2BM	Female	Spinal muscular atrophy Type 2	Positive; strong desire to reduce human assistance especially in case of simple actions in daily life (closing windows, lifting things, etc.); strong desire for mobility in daily life; underpinned the need for portable devices
F3BM	Female	Spinal muscular atrophy Type Kugelberg–Welander	Positive; strong desire to live independent; uses already several devices which allows her to drive car, do her work, etc.; concerns about the potential costs and demands that any technical support should not weaken the rest strength of her muscles
F4A	Female	Caregiver (unclear whether ‘at risk’ for ALS); her father had ALS (recently died)	Very positive; based on her experiences with own father she wishes better technical support for assisting communication; fear of being isolated without communication as ALS candidate, negative experiences with care in hospitals and with health insurance
M5BM	Male	ALS	Positive; especially toward non-invasive BCI; main interest is supporting and improving communication; strong concerns about invasive BCI related to his personal medical condition
M6BQ	Male	Tetraplegia	Ambivalent; positive attitudes toward technical devices in general; but concerns that BCI-developments will have high error-rates and personal support to manage them won’t be sufficiently provided
M7BM	Male	ALS	Positive; Strong interest in BCI, because he was not able to communicate clearly
M8BV	Male	Paraplegia after accident and possible error in treatment	Negative; fears adverse effects for rest mobility of the own body; prefers devices for rehabilitation or protection of still functioning parts of the body
F9BQ	Female	Paraplegia	Negative; strong concerns about adverse effects for the brain; worries that that BCI rather increases the need for human assistance because of her own medical condition; concerns about effects for the costs of daily care and the willingness of health insurances to cover costs for BCIs
M10BP	Male	Post-Polio-Syndrome	Ambivalent; positive toward technical progress; organizes his environment that he is able to act mostly independent; skeptical toward BCI because of unanswered questions how independent living in daily life is really possible

## Experts’ Positions Toward BCIs

In comparison to our findings of patients’ views, it is important to consider the major lines of arguments discussed by experts involved in the interdisciplinary assessment of BCI. Similar to many other biomedical innovations, such as stem cell research, xenotransplantation, or genetic profiling, the (justified) hopes and the (misleading) hypes are intensively discussed. This expert discourse can be seen as a socially necessary procedure of technology assessment and is often even politically initiated. Interestingly, readout-BCIs only very recently received considerable ethical attention. In the following, we will give a brief overview of the major lines of arguments as discussed in national reports, expert summary statements, and research articles by leading scholars. This unsystematic discursive analysis reveals the four lines of argumentation summarized in the following sections.

### Changes of Personality or Individual Identity

As the brain is seen as a major bodily correlate for our cognitive and emotional self-relationship, changes of the brain might lead to changes of a person’s identity. Some (e.g., Clark, 2004) use the metaphor ‘cyborg’ to express the transhumanist potential they expect from this technology. Other philosophers have used references to “cyborgs” to express concerns (e.g., Warwick, 2003; Rose, 2005; Wolpe, 2007) based on the idea that close interaction between the brain and a machine might impoverish our sensibility to differentiate between internal and external intentions and thus, our bodily awareness or consciousness can become prone to manipulation by external machines (Blanke and Aspell, 2009). Therefore, the neuro-invasive experiments by Delgado and others are considered a proof of concept for externally induced changes of one’s personal identity. However, the underlying assumption is here a Cartesian division of body and mind because the technical effect is imagined to be perceived as something ‘external’ intruding or an experience of estrangement. More holistic or dynamic approaches to the body and human identity refer to empirical evidence that external tools (BCIs, C-Leg prostheses, sports equipment etc.) are directly integrated into our body scheme. This can be seen as part of a successful body-environment interaction (Reichert et al., 2013) and therefore challenges the Cartesian view. The representation of the bodily self does not necessarily end at our body surface/outer skin but can or even must be extended to external tools (Nicolelis and Lebedev, 2009; Hildt, 2010). It is important to note that such a body conception is shared by modern neuroscience and postmodern ethics (Schicktanz, 2007). Some concerns toward BCIs derive from the assumption that a body is ‘natural’ and that an ‘unnatural’ technical system invades the human body. However, this view is inconsistent, as we accept many other forms of technological changes to the human body in the form of medical treatments (Anderson, 2008). According to Anderson only medical risks associated to BCIs or neuro-implants are relevant.

### Uncertainty About Risks

Several authors have pointed out that benefits and risks of BCIs are still uncertain for various reasons – the novelty of the approaches, a lack of comprehensive understanding of how the complex human brain works, and current challenges in data processing and control (Nuffield Council on Bioethics, 2013). The special status of the brain provides a reason to exercise beneficence by intervening when injury or illness causes brain disorders, and also a reason for caution when we are uncertain about the effects (Rose, 2005; Nuffield Council on Bioethics, 2013; Grübler et al., 2014). A radical ‘precautionary’ approach (Holm and Harris, 1999) might conclude that such uncertainty does not justify any research on BCIs. However, the implementation of such a principle necessitates caution, too, as it stifles any kind of research or discovery. Alternatively, a more procedural approach would allow for substantially distinguishing between invasive and non-invasive BCIs, as the former induce more medical risks than the latter. Moreover, we can work with different stages of careful risk assessment, as it is common for other clinical innovations (Clausen, 2009). This requires special scrutiny when informing patients and relatives about clinical trials. Overoptimistic promises or manipulation must be avoided during decision-making and consent processes (Hildt, 2010; Nuffield Council on Bioethics, 2013). This is a basic condition for any kind of biomedical innovation or research and *per se* is not specific for BCIs. In the case of BCIs, informed consent can be seen as a particular challenge for patients with both substantial impairment of mobility and verbal communication (Schneider et al., 2012), e.g., when involving patients with Locked-in-Syndrome. An argument for research on BCIs is that any opportunity should be taken enabling communication with such patients, while skeptics warn about wrong interpretations of a presumed will (Nijboer et al., 2009; Kyselo, 2013; Nijboer et al., 2013). However, considering most BCI technologies, the majority of the potential group of patients (e.g., with spinal cord injuries) are cognitively competent and are – in one way or another – able to communicate their will unambiguously. This is important to note, as readout-BCIs may in this context differ from other neuro-technologies such as neural tissue transplantations or brain stimulation for demented patients.

### Support for Family Members

A third ethically relevant argument in favor of BCIs for neuro-prosthesis is that it will particularly provide support for family members by reducing the burden of care (e.g., Nijboer et al., 2013). As BCIs in the context of neuro-prosthesis aims at gaining more independence for basic activities of patients such as moving, drinking, and eating, this can also improve the caregiver’s quality of life. While this is not wrong and a necessary part of clinical evaluation (Vaughan et al., 2012, p. 329f), overstressing such an argument can become problematic because it stigmatizes immobile patients as a burden for the family or society. It also overlooks that individual benefits for patients have ethical priority over such social considerations (UNESCO, 2005). Furthermore, it rather highlights how patients with impaired mobility still suffer from social and public stigmatization.

### BCIs as Neuro-Augmentation and Actual Enhancement

Several authors have hypothesized that neuro-technology cannot only restore impaired brains or body functions to “normal” brains but can also enhance them (Jebari, 2013; Nuffield Council on Bioethics, 2013; Earp et al., 2014). Whether such enhancement can be regarded as a morally acceptable aim is highly disputed within medical ethics (e.g., Buchanan, 2011). However, some authors (e.g., Attiah and Farah, 2014) criticized such a focus as a phantom debate. The authors argue that real enhancement is a hype since there is no actual evidence and call for more data. Moreover, the debate continues about what exactly “cognitive enhancement” means (see various contributions in Hildt and Franke, 2013). If we call any therapy enhancement in which the state is better than before, this might undermine the crucial differentiation between morally required and morally (in-)acceptable medical interventions.

In summary, in the field of bioethics and technology assessment, readout-BCIs are rather considered ‘science fiction’ than innovation close to clinical application. Readout-BCIs are often discussed in a broader range of other neuro-technologies such as neural tissue transplantation or even neuro-pharmaceuticals (Nuffield Council on Bioethics, 2013). This can lead to very general assumptions and tends to draw a rather indifferent picture of particular contexts. On the contrary, our results suggest that it is important to discuss the technological developments in their concrete context in order to receive sufficient specific assessments. In the following discussion we seek to elaborate on this argument and contrast experts’ and affected persons’ views.

## Discussion

Our study revealed practical and moral attitudes toward motor BCIs that can impact future technology development. Our sample’s focus on German patients and the chosen interview methodology require careful interpretation. The following considerations should therefore be understood as an early stage of hypothesis formation. These hypotheses can be then tested in future quantitative settings and in different cultural contexts. We will discuss four major issues (empowerment, changes in identity, enhancement, and technical risks) that emerged from the differences of how affected persons and experts assess and practically contextualize BCI technology.

### Empowerment

Both experts and patients consider autonomy and empowerment as crucial arguments. Consensually, communicative BCIs are seen as very important empowerment to allow patients to express their own wishes and needs. However, a closer comparative look reveals that their specific arguments might have different implications for BCI research. Experts have the tendency to believe that patients become autonomous by ‘walking again’ or by executing basic activities such as self-feeding. In addition, BCIs are seen as empowering for caregivers as they provides relief for their care work. Patients revealed a different perspective; they see BCIs as empowering for particular intimate activities and for allowing for more privacy in situations where dependence on human assistants is perceived as intrusive. In our problem-centered interviews patients put high priority on restoring bodily functions or daily activities related to body hygiene and intimacy. Our results are in accordance with previous studies (Hart et al., 1996; French et al., 2010; Hochberg and Anderson, 2012). These studies employed a structured survey with pre-formulated choices to assess spinal cord patients’ expectations toward BCIs. The authors found that these patients prioritize the restoration of bladder and bowel functions and sexual functions before, for example, regaining the ability to walk. The patients’ assessments indicate that autonomy and empowerment should be discussed from a more contextualized, disease-related perspective than from a theoretical, abstract one.

The topic of empowerment should also be regarded in its relation to the perception of disability. While the so called ‘deficit model’ (see above) is currently being heavily attacked by disability studies and accused of being morally loaded and promoting negative stereotypes (Pfeiffer, 2002), practical considerations do not neglect that persons having ALS, para- or tetraplegia need to be empowered in various forms to decide practically and politically about their own life. Thus, research on neuro-prosthesis for disabled persons should avoid hidden moral assumptions about the ‘deficits’ and rather focus on the interests of the affected persons themselves.

### Changes in Identity

Experts, especially from the field of philosophy, intensively discuss the issue of possible BCI-induced changes in identity. Aspects of consistency, stability, and authenticity of personal identity are indeed challenging philosophical questions. However, the current debate does not sufficiently differentiate between readout- and stimulation-BCIs as well as irreversible neuro-technologies. In contrast to experts, patients rarely problematize such issues when confronted with the BCI approach. This can be explained by the fact that most chronically ill patients have experienced radical changes of their identity and self-conception through their disease and disability (Strauss and Glaser, 1975; Kleinmann, 1988). A chronic disease can be understood from a sociological and psychological point as (painful) experience of identity transformation. In line with this view, the development of coping strategies in crises can be detected, as the patients continuously work on his/her identity. These changes often include the acceptance of dependence on technical or medical means to master everyday-life.

### Enhancement

A similar discrepancy between an expert debate driven by theory and an everyday-life perspective of patients can be observed with the issue of enhancement. In concrete applications it can be hard to decide whether an application is considered enhancement or therapy. For example, under the deficit hypothesis restoration of speech comprehension in CI-patients is considered therapy. If one does not consider deafness a deficit, CI would be considered as enhancement. This example shows that in concrete application situations the distinction depends on the assessment of the initial situation. If enhancement is understood broadly and relatively, it can be equated with learning (via or with technological support including book print or computers). Hence, it does not seem to be something new or problematic, but is seen as an integral part of human cultural evolution. However, if enhancement is understood narrowly as a technology that substantially changes the very bodily condition of a being, we might consider this intervention significant (e.g., creating human beings from their very existence with new genes for a different brain development). Also irreversibility seems to be important – if changes do not allow individuals to withdraw or change such a process, as we can do with other types of cognitive learning, a morally problematic intervention is implied. But the BCI technology we discussed above, does not fall into this first or second category. In this sense we are in line with Saniotis et al. (2014) who state that “while neuroscience research is advancing BMI therapeutic capabilities, there is yet no existing brain–machine interface based on exchange of electrical (electromagnetic) signals that would improve human cognitive abilities above and beyond what a natural brain can do.” Hence, it is reasonable that the interviewees did not refer to this philosophical debate.

### Technical Risks

Most obvious is that considerations shared by both the patients and professionals are related to technical and medical risks. Apparently, a proper risk assessment of prototypes of BCIs is required and necessary for the implementation in early phases of pilot studies and prototype development. Such risks also include particular disease-related medical risks, such as allergies, use of anaesthesia (in the case of invasive approaches), infections, and hazardous malfunctions. Patients were very concerned about medical and infectious risks related to invasive methods. Further risk studies with patients are needed to elaborate in detail acceptable risk-benefit trade-offs given the fact that invasive BCIs promise better performance.

## Conclusion

In the near future, readout-BCIs hopefully will provide a substantial breakthrough for neuro-prosthesis and neuro-rehabilitation. However, such clinical success will depend on the technology’s efficiency and the users’ acceptance. For the latter, it is crucial to avoid simple assumptions about disability such as a ‘medical deficit.’ Instead, disability needs to be understood in its social context including ease of communication, the relation between the patient and caregiver and the empowerment to meet everyday challenges. Most importantly, technology enabling more privacy and intimacy has high priority for patients with serious mobility impairment.

Thus far, with a few exceptions, research was has been conducted with healthy people. This allows for a proof of concept but risks missing the target groups’ interests. Currently, people of the target groups are mainly involved in later phases of clinical evaluation (Vaughan et al., 2012). However, our own explorative study, other existing surveys of patients’ perceptions (Anderson, 2004, 2009), and our review of expert opinions clearly indicate that there is a need for direct and deliberative interaction between the scientists as designers and developers of BCIs and the affected persons to develop user-centered designs that consider the user’s needs, desires, and abilities.

A suitable approach for this is the so-called “participatory design methodology”. Its basic premise consists of the idea of being “not *for* users, but *with* potential user tacit knowledge of their everyday-life” (see Spinuzzi, 2005; Clemensen et al., 2007). The basic concept of the participatory approach for technology development originated in Scandinavia in the 1970s. Below we will outline the steps of applying this concept of Spinuzzi (2005): First, an initial exploration of a disabled person’s life styles is performed (e.g., with protocols, ethnography (such as observations), or workshops with affected persons). In this step, information is acquired which goes beyond scientific-rational knowledge of a patient’s “deficities” and includes informal knowledge of the patient’s living situation, his/her coping strategies with everyday situations, frequency and type of social interactions, etc. This step helps to identify patients’ needs, desires, expectations and constraints for developing realistic scenarios, which can later be applied. The semi-structured interviews with BCI-inexperienced patients we conducted in our study can be seen as one approach for accomplishing this first step. Of course, more socio-empirical data would be needed to consider also culture-related acceptance. Second, understanding and prioritizing the needs, but again from both perspectives: the engineers’/scientists’ and the users’. Here, it is important to consider a larger number of users to identify the major priorities in needs. One example is technology enabling privacy and intimacy, which we have identified in our study; others (e.g., Widerström-Noga et al., 1999; Hochberg and Anderson, 2012) have identified related points. Third, prototyping in the lab and in real situations is necessary. The developed devices might be too cumbersome to handle or too sensitive to artifacts that are common in realistic environments (e.g., Grübler et al., 2014) and the commonly used paradigms might not be robust enough. In addition to engineering considerations, this process should ideally be guided by principles from a user-centered and cognitive design, as the specific user context and the cognitive burden of BCIs play an important role for its success. Furthermore, the results should not only be published in scientific journals but also be disseminated in a form (including everyday language) that can be understood by the potential users. This is most important for an adequate process of evaluation of the approach.

Of course, there are also limitations for such research. First of all, a participatory design is sophisticated and requires an interdisciplinary setting (Clemensen et al., 2007; Shah and Robinson, 2007). Especially social scientists are often better trained for implementing methodologies of ethnography, user participation, or for overcoming language gaps between experts and lay persons. Therefore, engineers, neuroscientists, clinicians, and social scientists need to collaborate to identify the target groups and to involve them actively. Participatory methodology as a research approach is time and money consuming, as stage 1 and 2 especially take considerably more effort. However, in the long run, it is very likely that such an approach will be more efficient and will avoid extensive and expensive technology development for, a very small user group.

## References

[B1] Anderson J. (2008). “Neuro-Prosthetic, the extended mind and respect for persons with disability,” in *The Contingent Nature of Life. Bioethics and Limits of Human Existence*, eds Düwell M. Rehmann-Sutter C. Mieth D. (Dordrecht: Springer), 259–275.

[B2] Anderson K. D. (2004). Targeting recovery: priorities of the spinal cord injured population. *J. Nerotrauma* 21 1371–1383 10.1089/neu.2004.21.1371 15672628

[B3] Anderson K. D. (2009). Consideration of user priorities when developing neural prosthetics. *J. Neural. Eng.* 6:5 10.1088/1741-2560/6/5/055003 19721182

[B4] Attiah M. A. Farah M. J. (2014). Minds, motherboards, and money: futurism and realism in the neuroethics of BCI technologies. *Front. Syst. Neurosci.* 8:86 10.3389/fnsys.2014.00086 PMC4030132 24860445

[B5] Beck U. (2000). “Risk society revisited. Theory, politics and research programmes,” in *The Risk Society and Beyond*, eds Adam B. Beck U. Van Loost J. (London: Sage), 211–229.

[B6] Birbaumer N. Ghanayim N. Hinterberger T. Iversen I. Kotchoubey B. Kübler A. (1999). A spelling device for the paralysed. *Nature* 398 297–298 10.1038/18581 10192330

[B7] Blanke O. Aspell J. E. (2009). Brain technologies raise unprecedented ethical challenges. *Nature* 458:703 10.1038/458703b 19360064

[B8] Blume S. (2009). *The Artificial Ear: The Cochlea Implant and the Culture of Deafness*. New Brunswick: Rutgers University Press.

[B9] Buchanan A. (2011). *Beyond Humanity?: The Ethics of Biomedical Enhancement*. New York, NY: Oxford University Press 10.1093/acprof:oso/9780199587810.001.0001

[B10] Carlson T. del Millan R. J. (2013). Brain–controlled wheelchairs: a robotic architecture. *IEEE Robot. Autom. Mag.* 20 65–73 10.1109/MRA.2012.2229936

[B11] Clark A. (2004). *Natural-Born Cyborgs*. New York, NY: Oxford University Press.

[B12] Clark G. M. (2014). The multi-channel cochlear implant: multi-disciplinary development of electrical stimulation of the cochlea and the resulting clinical benefit. *Hear. Res*. 322 4–13 10.1016/j.heares.2014.08.002 25159273

[B13] Clausen J. (2009). Man, machine and in between. *Nature* 457 1080–1081 10.1038/4571080a 19242454

[B14] Clemensen J. Larsen S. B. Kyng M. Kirkevold M. (2007). Participatory design in health sciences. Using cooperative experimental methods in developing health service and computer technology. *Qual. Health Res.* 17 122–130 10.1177/1049732306293664 17170250

[B15] Dadarlat M. C. O’Doherty J. E. Sabes P. N. (2015). A learning-based approach to artificial sensory feedback leads to optimal integration. *Nat. Neurosci.* 18 138–144 10.1038/nn.3883 25420067 PMC4282864

[B16] Delgado J. M. R. (1969). *Physical Control of the Mind: Toward a Psychocivilized Society*. New York, NY: Harper & Row.

[B17] Earp B. D. Sandberg A. Kahane G. Savulescu J. (2014). When is diminishment a form of enhancement? Rethinking the enhancement debate in biomedical ethics. *Front. Syst. Neurosci.* 8:12 10.3389/fnsys.2014.00012 PMC3912453 24550792

[B18] Fitzsimmons N. Lebedev M. A. Peikon I. Nicolelis M. A. L. (2009). Extracting kinematic parameters for monkey bipedal walking from cortical neuronal ensemble activity. *Front. Integr. Neurosci.* 3:1–19 10.3389/neuro.07.003.2009 19404411 PMC2659168

[B19] French J. S. Anderson-Erisman K. D. Sutter M. (2010). What do spinal cord Injury consumers want? A review of spinal cord injury consumer priorities and neuroprosthesis from the 2008 neural interfaces conference. *Neuromodulation* 13 229–231 10.1111/j.1525-1403.2009.00252.x 21992837

[B20] Grübler G. Al-Khodairy A. Leeb R. Pisotta I. Riccio A. Rohm M. (2014). Psychosocial and ethical aspects in non-invasive EEG-based BCI research—A survey among BCI users and BCI professionals. *Neuroethics* 7 29–41 10.1007/s12152-013-9179-7

[B21] Guger C. Brendan Z. Edlinger G. (eds). (2013). *Brain–computer interface Research: A State-of-the- (Art )Summary*. Dordrecht: Springer 10.1007/978-3-642-36083-1

[B22] Hart K. A. Rintala D. H. Fuhrer M. J. (1996). Educational interests of individuals with spinal cord injury living in the community: medical, sexuality, and wellness topics. *Rehabil. Nurs.* 21 82–90 10.1002/j.2048-7940.1996.tb01681.x 8701099

[B23] Hess W. R. (1932). *Beiträge zur Physiologie des Hirnstammes. 1. Die Methodik der Lokalisierten Reizung und Ausschaltung Subkortikaler Hirnabschnitte*. Leipzig: Thieme.

[B24] Hildt E. (2010). Brain–computer interaction and medical brain–computer interaction and medical access to the brain: individual, social and ethical implications ethical implications. *Stud. Ethics Law Technol.* 4:3 10.2202/1941-6008.1143

[B25] Hildt E. Franke A. (eds) (2013). *Cognitive Enhancement: An Interdisciplinary Perspective*. Dordrecht: Springer 10.1007/978-94-007-6253-4

[B26] Hochberg L. Anderson K. (2012). “BCI users and their needs,” in *Brain–Computer Interfaces: Principles and practice*, eds Wolpaw J. Wolpaw E. W. (New York, NY: Oxford University Press), 317–324 10.1093/acprof:oso/9780195388855.003.0019

[B27] Hochberg L. R. Bacher D. Jarosiewicz B. Masse N. Y. Simeral J. D. Vogel J. (2012). Reach and grasp by people with tetraplegia using a neurally controlled robotic arm. *Nature* 485 372–375 10.1038/nature11076 22596161 PMC3640850

[B28] Hollmann M. Rieger J. W. Baecke S. Lützkendorf R. Müller C. Adolf D. (2011). Predicting decisions in human social interactions using real-time fMRI and pattern classification. *PLoS ONE* 6:e25304 10.1371/journal.pone.0025304 PMC3189203 22003388

[B29] Holm S. Harris J. (1999). Precautionary principle stifles discovery. *Nature* 400 398 10.1038/22626 10440359

[B30] Huggins J. E. Wren P. A. Gruis K. L. (2011). What would brain–computer interface users want? Opinions and priorities of potential users with amyotrophic lateral sclerosis. *Amyotroph. Lateral Scler.* 12 318–324 10.3109/17482968.2011.572978 21534845 PMC3286341

[B31] Jebari K. (2013). Bonding brains to machines: ethical implications of electroceuticals for the human brain. *Neuroethics* 6 429–434 10.1007/s12152-013-9186-8

[B32] Kleinmann A. (1988). *The Illness Narratives: Suffering. Healing and the Human Condition*. New York, NY: Basic Books.

[B33] Kotsch L. (2012). *Assistenzinteraktionen. Zur Interaktionsordnung in der Persönlichen Assistenz Körperbehinderter Menschen*. Wiesbaden: Springer VS 10.1007/978-3-531-93155-5

[B34] Kringelbach M. L. Jenkinson N. Owen S. L. F. Aziz T. Z. (2007). Translational principles of deep brain stimulation. *Nat. Rev. Neurosci.* 8 623–635 10.1038/nrn2196 17637800

[B35] Kübler A. Nijboer F. Mellinger J. Vaughan T. M. Pawelzik H. Schalk G. (2005). Patients with ALS can use sensorimotor rhythms to operate a brain–computer–interface. *Neurology* 64 1775–1777 10.1212/01.WNL.0000158616.43002.6D 15911809

[B36] Kyselo M. (2013). Locked-in syndrome and BCI - towards an enactive approach to the self. *Neuroethics* 6 579–591 10.1007/s12152-011-9104-x

[B37] Lebedev M. A. Nicolelis M. A. L. (2006). Brain–machine interfaces: past, present and future. *Trends Neurosci.* 29 536–546 10.1016/j.tins.2006.07.004 16859758

[B38] Lebedev M. A. Tate A. J. Hanson T. L. Li Z. O’Doherty J. E. Winans J. A. (2011). Future developments in brain-machine interface research. *Clinics* 66(Suppl. 1), 25–32 10.1590/S1807-59322011001300004 21779720 PMC3118434

[B39] Mayring P. (2010). *Qualitative Inhaltsanalyse. Grundlagen und Techniken*. Weinheim: Beltz.

[B40] Nicolelis M. A. L. Lebedev M. A. (2009). Principles of neural ensemble physiology underlying the operation of brain–machine interfaces. *Nat. Rev. Neurosci.* 10 530–540 10.1038/nrn2653 19543222

[B41] Nijboer F. Clausen J. Allison B. Z. Haselager P. (2013). The asilomar survey. Stakeholders’ opinions on ethical issues related to brain–computer–interfacing. *Neuroethics* 6 541–578 10.1007/s12152-011-9132-6 24273623 PMC3825606

[B42] Nijboer F. Kleih S. Kübler A. (2009). “Gehirn-Computer-Schnittstellen für schwerstgelähmte Menschen. Klinische Möglichkeiten, technische Grenzen und ethische Fragen,” in *Das technisierte Gehirn. Neurotechnologien als Herausforderung für Ethik und Anthropologie*, eds Müller O. Clausen J. Maio G. (Paderborn: Mentis), 51–62.

[B43] Nijboer F. Sellers E. W. Mellinger J. Jordan M. A. Matuz T. Furdea A. (2008). A P300-based brain–computer–interface for people with amytrophic lateral sclerosis. *Clin. Neurophysiol.* 119 1909–1916 10.1016/j.clinph.2008.03.034 18571984 PMC2853977

[B44] Nuffield Council on Bioethics. (2013). *Novel Neurotechnologies: Intervening in the Brain.* London: Nuffield Council on Bioethics.

[B45] O’Doherty J. E. Lebedev M. A. Ifft P. J. Zhuang K. Z. Shokur S. Bleuler H. (2011). Active tactile exploration using a brain–machine–brain interface. *Nature* 479 228–231 10.1038/nature10489 21976021 PMC3236080

[B46] Onose G. Grozea C. Anghelescu A. Daia C. Sinescu C. J. Ciurea A. V. (2012). On the feasibility of using motor imagery EEG-based brain–computer interface in chronic tetraplegics for assistive robotic arm control: a clinical test and long-term post-trial follow-up. *Spinal Cord* 50 599–608 10.1038/sc.2012.14 22410845

[B47] Pfeiffer D. (2002). The philosophical foundations of disability studies. *Disabil. Stud. Q.* 22 3–23.

[B48] Quandt F. Reichert C. Schneider B. Dürschmid S. Richter D. Hinrichs H. (2012a). Fundamentals and application of brain-machine interfaces. *Klin. Neurophysiol.* 43 158–167 10.1055/s-0032-1308970

[B49] Quandt F. Reichert C. Hinrichs H. Heinze H. J. Knight R. T. Rieger J. W. (2012b). Single trial discrimination of individual finger movements on one hand: a combined MEG and EEG study. *Neuroimage* 59 3316–3324 10.1016/j.neuroimage.2011.11.053 22155040 PMC4028834

[B50] Reichert C. Fendrich R. Bernarding J. Tempelmann C. Hinrichs H. Rieger J. W. (2014). Online tracking of the contents of conscious perception using real-time fMRI. *Front. Neurosci.* 8:116 10.3389/fnins.2014.00116 PMC4033165 24904260

[B51] Reichert C. Kennel M. Kruse R. Heinze H.-J. Schmucker U. Hinrichs H. (2013). “Robotic grasp initiation by gaze independent brain-controlled selection of virtual reality objects. NEUROTECHNIX 2013” in *Proceedings of the International Congress on Neurotechnology, Electronics and Informatics*, Vilamoura 5–12.

[B52] Rieger J. W. Reichert C. Gegenfurtner K. R. Noesselt T. Braun C. Heinze H.-J. (2008). Predicting the recognition of natural scenes from single trial MEG recordings of brain activity. *Neuroimage* 42 1056–1068 10.1016/j.neuroimage.2008.06.014 18620063

[B53] Rose S. (2005). *The Future of the Brain. The Promise and Perils of Tomorrow’s Neuroscience*. New York, NY: Oxford University Press.

[B54] Saniotis A. Henneberg M. Kumaratilake J. Grantham J. P. (2014). “Messing with the mind”: evolutionary challenges to human brain augmentation, *Front. Syst. Neurosci.* 8:152 10.3389/fnsys.2014.00152 PMC4179735 25324734

[B55] Schicktanz S. (2007). Why the way we consider the body matters. Reflections on four bioethical perspectives on the human body. *Philos. Ethics Humanit. Med.* 2:30 10.1186/1747-5341-2-30 PMC2180175 18053201

[B56] Schneider M.-J. Fins J. J. Wolpaw J. R. (2012). “Ethical issues in BCI-research,” in *Brain–Computer Interfaces. Principles and Practice*, eds Wolpaw J. R. Wolpaw E. W. (New York, NY: Oxford University Press), 373–383.

[B57] Shah S. G. S. Robinson I. (2007). Benefits of and barriers to involving users in medical device technology development and evaluation. *Int. J. Tech. Assess. Health Care* 23 131–137 10.1017/S0266462307051677 17234027

[B58] Snoek G. J. Ijzerman M. J. Hermens H. J. Maxwell D. Biering-Sorensen F. (2004). Survey of the needs of patients with spinal cord injury: impact and priority for improvement in hand function in tetraplegics. *Spinal Cord* 42 526–532 10.1038/sj.sc.3101638 15224087

[B59] Spinuzzi C. (2005). The Methodology of participatory design. *Tech. Commun.* 52 163–174.

[B60] Strauss A. Glaser B. (1975). *Chronic Illness and the Quality of Life*. St. Louis: C.V. Mosby Publishing.

[B61] Svirsky M. A. Teoh S.-W. Neuburger H. (2004). “Development of language and speech perception in congenitally, profoundly deaf children as a function of age at cochlear implantation.” *Audiol. Neurootol.* 9 224–233 10.1159/000078392 15205550

[B62] UNESCO. (2005). *Universal Declaration of Bioethics and Human Rights*. Available at: http://portal.unesco.org/en/ev.php-URL_ID=31058&URL_DO=DO_TOPIC&URL_SECTION=201.htmlLatest [access January 28, 2015]. 17139812

[B63] Vaughan T. M. Sellers E. W. Wolpaw J. R. (2012). “Clinical Evaluation of BCI,” in *Brain–Computer Interfaces. Principles and Practice*, eds Wolpaw J. R. Wolpaw E. W. (New York, NY: Oxford University Press), 325–335.

[B64] Velliste M. Perel S. Spalding M. C. Whitford A. S. Schwartz A. B. (2008). Cortical control of a prosthetic arm for self-feeding. *Nature* 453 1098–1110 10.1038/nature06996 18509337

[B65] Vidal J. J. (1973). Toward Direct Brain–Computer Communication. *Annu. Rev. Biophys. Bioeng.* 2 157–180 10.1146/annurev.bb.02.060173.001105 4583653

[B66] Warwick K. (2003). Cyborg morals, cyborg values, cyborg ethics. *Ethics Inf. Technol.* 5 131–137 10.1023/B:ETIN.0000006870.65865.cf

[B67] Widerström-Noga E. G. Felipe-Cuervo E. Broton J. G. Duncan R. C. Yezierski R. P. (1999). Perceived difficulty in dealing with consequences of spinal cord injury. *Arch. Phys. Med. Rehabil*. 80 580–586 10.1016/S0003-9993(99)90203-4 10326925

[B68] Witzel A. Reiter H. (2012). *The Problem-Centred Interview*. London: SAGE.

[B69] Wolpaw J. R. Wolpaw E. W. (Eds) (2012). *Brain–computer interfaces: Principles and Practice*. New York, NY: Oxford University Press 10.1093/acprof:oso/9780195388855.001.0001

[B70] Wolpe P. R. (2007). Ethical and social challenges of Brain–Computer–Interfaces. *Virtual Mentor* 9 128–131 10.1001/virtualmentor.2007.9.2.msoc1-0702 23217761

